# Fish Bones as Calcium Source: Bioavailability of Micro and Nano Particles

**DOI:** 10.3390/foods13121840

**Published:** 2024-06-12

**Authors:** Benjamín Torres, Alvaro Pérez, Paula García, Paula Jiménez, Karen Abrigo, Pedro Valencia, Cristian Ramírez, Marlene Pinto, Sergio Almonacid, Manuel Ruz

**Affiliations:** 1 Department of Nutrition, Faculty of Medicine, Universidad de Chile, Santiago 8380453, Chile; benjamin.torres@inta.uchile.cl (B.T.); afperezb@uchile.cl (A.P.); pgarcia@uchile.cl (P.G.); paulajimenez@uchile.cl (P.J.); kabrigo.blanc@gmail.com (K.A.); 2 Centro de Biotecnología Daniel Alkalay Lowitt, Universidad Técnica Federico Santa María, Valparaíso 2390136, Chile; pedro.valencia@usm.cl; 3 Departamento de Ingeniería Química y Ambiental, Universidad Técnica Federico Santa María, Valparaíso 2390123, Chile; cristian.ramirez@usm.cl (C.R.); marlene.pinto@usm.cl (M.P.); sergio.almonacid@usm.cl (S.A.)

**Keywords:** calcium, fish, bone, nanoparticles, bioavailability, mice

## Abstract

The amount of by-products/waste in the fish industry is roughly 50%. Fish bones could be used to produce nanoparticles, which may have potential use in the food industry as a novel calcium source and at the same time, contribute to reduce waste production. The objective of this study was to evaluate the bioavailability of nano-size salmon fish bone particles compared to micro-size salmon fish bone particles, and calcium carbonate. The study was carried out in 21–28-day-old C57BL/6 male mice fed for 21 days with the experimental diets. The groups were as follows: CaCO_3_ 0.5% Ca (CN 0.5); CaCO_3_ 1.0% Ca (CN 1.0); salmon fish bone (SFB) microparticles 0.5% Ca (MP 0.5); SFB microparticles 1.0% Ca (MP 1.0); SFB nanoparticles 0.5% Ca (NP 0.5); and SFB nanoparticles 1.0% Ca (NP 1.0). Calcium bioavailability, defined as the percent calcium in femur showed an increasing trend from CN 0.5 to NP 1.0 group. According to ANCOVA, the greatest Ca content was observed in the NP 1.0 group compared with all groups but NP 0.5. In conclusion, in a murine model, salmon fish bone nanoparticles present higher calcium bioavailability than salmon fish bone microparticles, and both, in turn, have better bioavailability than calcium carbonate.

## 1. Introduction

Currently, to address an issue dealing with the interface of nutrition and foods, it is crucial to set it in a broader context, such as sustainable development. As a way to operationalize it, the concept of circular economy becomes instrumental, although there is no single definition accepted [1]. Among these, that suggested by van Buren et al. [2] probably suits the purpose of the topic developed in this study best. This definition states that a circular economy aims at the creation of economic value (i.e., the economic value of the products increases), the creation of social value (for example, the prevention of unhealthy working conditions in the extraction of raw materials and reuse), and also the creation of value in terms of the environment (such as reducing waste production).

In 2020, the estimated global fishery and aquaculture production reached approximately 214 million tons [3]. The amount of by-products in the fish industry is roughly 50% [4]. By-products refer to components that are discarded, such as viscera, heads, bone, cut-offs, skin, and fish that are damaged or unsuitable for human consumption [4]. In order to reduce the environmental impact of fish processing, in terms of waste reduction, the industry utilizes by-products in the production of silage or the use of waste in the manufacture of organic fertilizer and fishmeal/oil [5].

The primary product of the fish industry is fish fillet. Removing inedible components also improves the value/unit and reduces transportation cost. Fish frames are the leftovers from fish fillet production, and they consist of protein, lipid, and bones [6]. In turn, fish bone is mainly composed of minerals such as calcium phosphate and proteins that can be used as ingredients in the food industry [7]. Fish bones have great potential as a source of bioavailable calcium. However, technological aspects related to calcium extraction and solubilization are of great importance in increasing calcium concentration and bioavailability [8]. These aspects deserve special attention.

Calcium is the most abundant inorganic nutrient in the human body, approximately 1.2–1.6 kg in an adult. Approximately 99% of calcium is in the skeleton and bones; the remaining 1% is in fluids and soft tissue, performing critical roles in enzyme activities, coagulation, hormonal signaling, neuronal communication, muscular contraction, and cell signaling. Blood calcium is tightly controlled at the intestine, kidney, and bone levels by hormones such as calcitonin and parathormone [9,10]. A recent systematic review found that calcium intake is very low in many countries of the Asia–Pacific region (<400 mg/day) and South America (400–600 mg/day), including Chile; just a few countries in Northern Europe have an average calcium intake greater than the recommended 1000 mg/day [9,11,12]. Only a portion of calcium ingested is absorbed and retained, approximately 25–30% [13]. However, this can vary in a wide range, affected by endogenous and exogenous factors. Among endogenous factors, life stage, hormonal status, and the presence of certain diseases can be included. Among exogenous factors, dietary components are highly relevant, such as lactose, proteins, minerals, oxalic acid, phytic acid, and some pharmacologic products [14,15].

Recently, nanotechnology has been explored as an alternative to improve the bioavailability of nutrients and nutraceuticals [16,17]. Nanoparticles can be generated either through a mechanical process or the assembly of smaller molecules, such as lipids and proteins, in a chemical process [18]. Although the mechanisms involved are not fully understood, an increase in the apparent solubility of the active ingredient and in direct uptake may take place [18]. In this context, fish bones could be used to produce nanoparticles, which may have potential use in the food industry as a novel, highly bioavailable calcium source and, at the same time, contribute to reducing waste production.

The objective of this study was to evaluate the bioavailability of nano-size salmon fish bone particles compared to micro-size salmon fish bone particles, and calcium carbonate, a common product used as a supplement and in the food industry.

## 2. Materials and Methods

### 2.1. Animals

The study was carried out in 21–28 days old C57BL/6 male mice obtained from the Central Animal Facility of the Faculty of Medicine University of Chile. Animals were allowed to adapt to a powdered diet for 7–14 days and then randomly assigned to 6 experimental groups. All animals were fed a calcium-free AIN93 G diet obtained from Research Diets (New Brunswick, NJ, USA), as a base diet with variations only in Ca source and concentration (at 0.5% and 1%); the former agrees with the dietary calcium requirement [19], and the latter may resemble levels used in supplementation or fortification. The experimental groups were as follows. Control diets contained calcium carbonate, CaCO_3_, as calcium source, which is customarily used in rodent diets: CaCO_3_ 0.5% Ca (CN 0.5); CaCO_3_ 1.0% Ca (CN 1.0); salmon fish bone (SFB) microparticles 0.5% Ca (MP 0.5); SFB microparticles 1.0% Ca (MP 1.0); SFB nanoparticles 0.5% Ca (NP 0.5); and SFB nanoparticles 1.0% Ca (NP 1.0). The animals were maintained on the diets fed ad libitum during 21 days at the Department of Nutrition Animal Facility in a 12 h light/dark cycle at 22 ± 2 °C and with free access to deionized water. After treatment, animals were anesthetized with a combination of ketamine/xylazine (90/10 mg/kg BW) and sacrificed by exsanguination through cardiac puncture; back legs were obtained, and after skin and muscle removal, femurs were processed for ash and calcium determinations.

**Figure 1:**
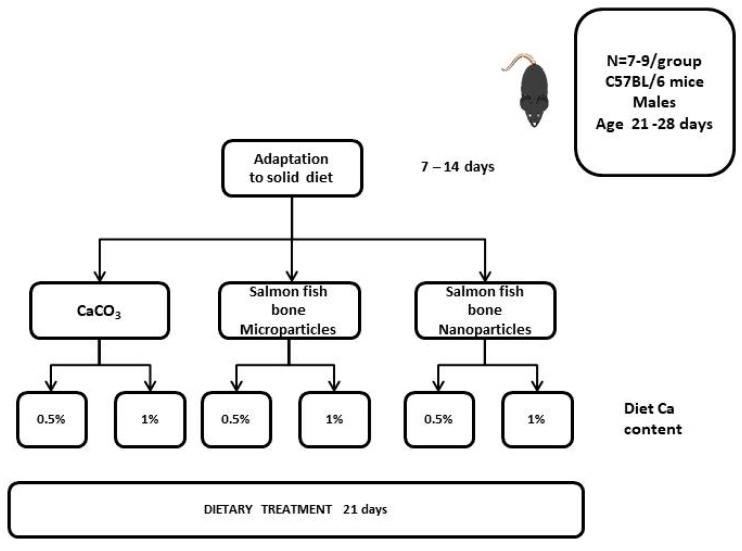
Scheme of the experimental design.

A schematic representation of the experimental design is in Figure 1.

The protocol of this study was approved by the University of Chile, Institutional Committee for Use and Care of Animals (CICUA), protocol CBA 1232 FMUCH.

### 2.2. Procedures and Methods

Salmon frames were cut and ground to produce a slurry paste. This was followed by the enzymatic hydrolysis of salmon-frame proteins. These procedures have been published in detail elsewhere [20,21]. Briefly, this was carried out in an agitated batch reactor where the ground salmon frame was mixed with 13 AU of subtilisin, an endoproteinase from *Bacillus lichenoformis* supplied by Novozymes (Bagsvaerd, Denmark). The operational conditions were at 55 °C and pH 6.5. After the operation of the batch reactor, the reaction mixture was centrifuged. This is referred to as salmon frame protein hydrolysate. The solid phase (bone) generated from the enzymatic hydrolysis was separated by decanting and dried (105 °C for 6 h in an oven), obtaining small, dried flakes, and kept until used to produce micro and nano salmon fish bones particles. The dried bone was subjected to coarse milling for 5 min using a pulverizer, to obtain a bone powder. A planetary ball media mill (Retsch, PM-100, Haan, Germany) was used for further dry and wet milling processing. The particle size (d_50_ and distribution) is a function of four main parameters: rotation speed, ball-media/powder weight ratio, media diameter, and milling time. A fifth parameter is added when wet milling is carried out; powder/water weight ratio. Thus, a d_50_ particle size of 10 µm, referred to as salmon fish bone microparticle (MP), was obtained by dry milling. A stainless-steel milling jar (250 mL) and balls of 10 mm in diameter (same material) were used. The process was conducted at 400 rpm, 4:1 ball-media/bone-powder weight ratio, and 10 min grinding time. Similarly, since particles below 1 µm tend to aggregate, the d_50_ = 0.25 µm particle size was obtained by wet milling. A stainless-steel milling jar (250 mL) and balls of 1 mm in diameter (same material) were used. The process was conducted at 400 rpm, 10:1 ball-media/bone-powder weight ratio, 20/80 bone-powder/water weight ratio, and 2.5 h grinding time. This is referred to as salmon fish bone nanoparticle (NP). The particle size distribution and corresponding d_50_ were determined with a mastersizer (Dandong Bettersize Instruments Ltd., BT-9300H, Qingdao, China) for microparticles and with a nanosizer (Malvern Instruments Ltd., Zetasizer Nano ZS90, Malvern, UK) for nanoparticles. Experimental particle size determinations were carried out in triplicate; results were as follows: microparticles d_50_ = 10.54 ± 0.19 µm, nanoparticles d_50_ = 0.25 ± 0.013 µm, calcium carbonate used in control groups d_50_ = 0.75 ± 0.070 µm. Calcium content (on dry basis) were 20.8 ± 1.85 g/100 g in microparticles and 22.4 ± 3.66 g/100 g in nanoparticles. Determinations were conducted using atomic absorption spectrophotometry.

### 2.3. Determinations in Experimental Animals

After sacrifice, animals were dissected. Skin and muscle were removed from back legs. Thus, femur was available for further processing and analysis.

Ash and calcium determination in mice femur: After femur removal, they were cleaned to eliminate remaining protein, and then length was measured using a digital caliper. Femur bone was milled in a mortar, and dried at 105 °C until weight was stable. Bone powder was transferred to crucibles and maintained during 8 h at 500 °C in a muffle furnace. Ashes were weighed and digested by adding 2 mL concentrated trace element grade nitric acid (Fisher Scientific, Waltham, MA, USA), which was maintained until dryness. Samples were kept during 1 h in a muffle furnace at 500 °C. Later, 10 mL 1 N HCl acid suprapur grade (Merck KGaA, Darmstadt, Germany) was added. These solutions were diluted with deionized water and 0.1% lanthanum nitrate solution. Calcium determinations were carried out in a Perkin Elmer AAnalyst-100 atomic spectrophotometer using the flame mode.

Statistical analyses were carried out using SPSS 21 software (IBM SPSS statistics, Armonk, NY, USA). A probability value < 0.05 was considered significant.

## 3. Results

### 3.1. General Characteristics of Experimental Animals

In Table 1, the weight of animals before the administration of experimental diets, food intake during the 21 days of dietary treatment, and body weight, femur weight, and femur length after the experimental period are presented. As explained in the Materials and Methods section, animals of 21–28 days of age were obtained from the Faculty of Medicine animal facility and later subjected to a period of adaptation to a solid diet of 7–14 days. As a result of the range in age and the adaptation period, some differences in initial weight were observed, as indicated in the table. Since this may have an effect on parameters related to the objective of the study, former comparisons of results in bone ash and bone calcium concentrations were conducted with this variable treated as a covariable. Regarding femur weight, the NP 0.5 and NP 1.0 groups showed the highest values and the CN 0.5 group the lowest. Dissimilarities in femur length among groups were less marked, although some statistical differences were also noted.

### 3.2. Results in Mouse Bone

**Figure 2:**
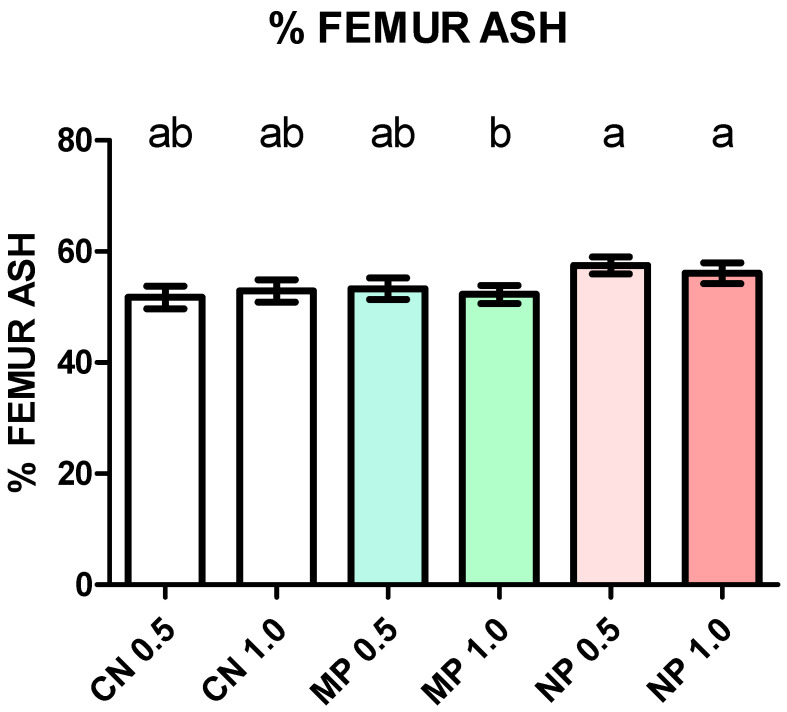
Femur ash in mice after 21 days of dietary treatment with diets varying in calcium source and concentration. Values are mean ± SD. Values represent percent of ash in relation to bone weight on dry basis. Statistical analysis by analysis of covariance (ANCOVA), initial weight as covariable (*p* = 0.565), group effect *p* < 0.004. Different letters on top of bars are significant differences according to Bonferroni’s post hoc test (*p* < 0.05).

In order to have a gross estimation of mineral concentration in the femur, % femur ash was determined, and results are presented in Figure 2. ANCOVA showed minor variations. The values of NP 0.5 (57.5 ± 1.55) and NP 1.0 (56.1 ± 1.86) were significantly higher than those of MP 1.0 (52.3 ± 1.63) but not MP 0.5 (53.3 ± 1.93) and controls.

**Figure 3:**
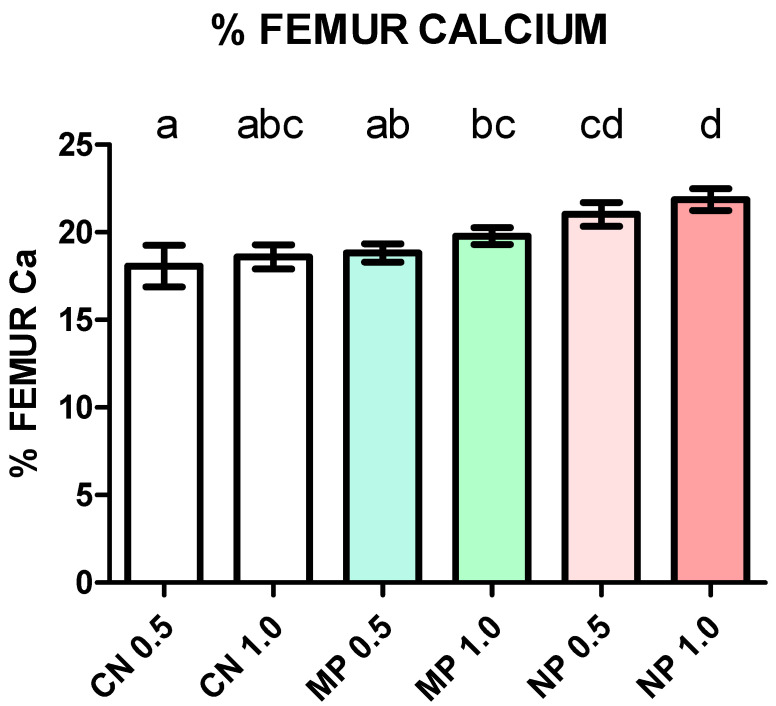
Femur calcium concentration in mice after 21 days of dietary treatment with diets varying in calcium source and concentration. Values are mean ± SD. Values represent percent of calcium in relation to bone weight on dry basis. Statistical analysis by analysis of covariance (ANCOVA), initial weight as covariable (*p* = 0.165), group effect *p* < 0.0001. Different letters on top of bars are significant differences according to Bonferroni’s post hoc test (*p* < 0.05).

Percent Ca represents Ca retention in bone and is, in turn, a measure of Ca bioavailability. These results are presented in Figure 3. A visual inspection of graph bars shows an increasing gradient from the CN 0.5 to the NP 1.0 group. Mean ± SD (as %) were 18.1 ± 1.19, 18.6 ± 0.68, 18.8 ± 0.52, 19.8 ± 0.48, 21.0 ± 0.67, and 21.9 ± 0.6, correspondingly. According to ANCOVA, the greatest Ca content was observed in the NP 1.0 group compared with all groups but NP 0.5.

**Figure 4:**
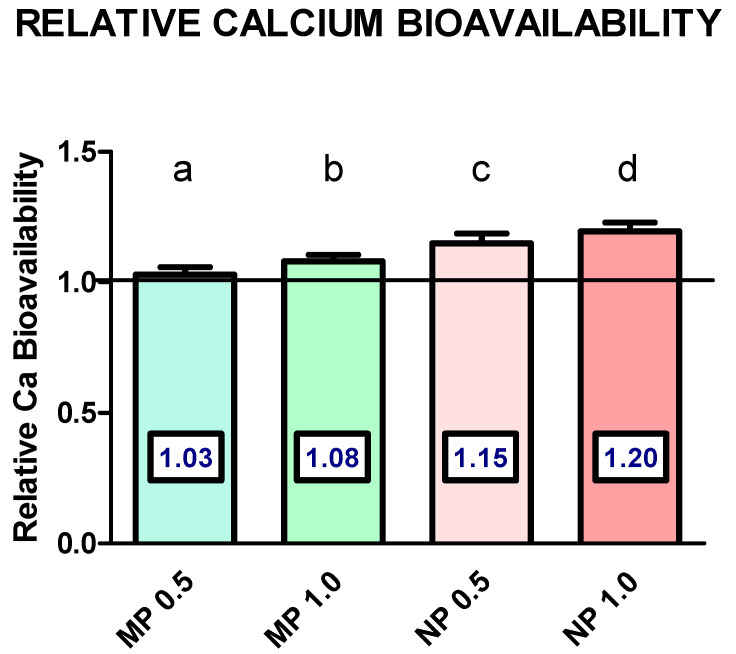
Relative calcium bioavailability in mice after 21 days of dietary treatment with diets varying in calcium source. Values are mean ± SD. Values are expressed as % femur Ca of each treatment relative to % femur Ca in the CN 0.5 and CN 1.0 groups combined (horizontal line). Statistical analysis by one-way ANOVA. Different letters on top of bars are statistically different according to Tukey’s post hoc test (*p* < 0.05).

Figure 4 shows relative calcium bioavailability expressed as ratios between % femur Ca in each group in relation to an average of % femur Ca obtained in the CN 0.5 and CN 1.0 groups combined because they did not differ in % Ca content, as seen in the previous figure. This was considered as value = 1. Results presented in this form allow for an alternative and clearer way to assess the relative Ca bioavailability of experimental groups. Thus, relative bioavailability could be interpreted as follows. Relative calcium bioavailability in salmon fish bone microparticle groups at 0.5% and 1.0% in the diet is 3% and 8% greater than controls, respectively. Relative calcium bioavailability in salmon fish bone nanoparticle groups at 0.5% and 1.0% in the diet is 15% and 20% greater than controls, respectively. All differences reached statistical significance.

## 4. Discussion

Calcium intake is the main determinant of calcium status. Calcium deficiency is not limited to altered bone health, but it may also be related to other health outcomes, such as hypertension, hypercholesterolemia, and some forms of cancer [22]. The Nutrition Science program of the New York Academy of Science published a study on worldwide calcium deficiency. Its main findings were that people from low- and middle-income countries are at greater risk of inadequate calcium intake, and that individuals from high-income countries are not exempt from this condition [23]. This is consistent with a recent review in women from low- and middle-income countries [24] who presented calcium intakes below the estimated adequate requirement (EAR) of 800 mg/d in 13 out of the 14 countries studied [9]. In Chile, where the present study was conducted, the National Food Consumption Survey showed that calcium intake in adults is 40–50% of the EAR [12]. This situation is a driving force for finding new calcium sources. Fish bones as a potential calcium source have been limited explored [8,25].

Typically, discarded fish bones represent about 9% to 15% of the weight of the whole fish and are composed of 30% to 45% protein, 15% to 60% ash, mainly calcium, and less fat content depending on the type of fish [8]. The enzymatic hydrolysis of the fish frames makes it possible to eliminate the muscle and connective tissue that remain attached to the bones of the fish, recover a significant amount of high-value peptides, and concentrate calcium [20,21]. After hydrolysis with subtilisin, the fish bone micro- and nanoparticles had almost 20% calcium (on a dry matter basis), which is expected in clean fish bones. This calcium content is slightly lower than calcium carbonate (40% calcium) but is similar to other calcium sources commonly used in the food industry, such as calcium citrate (24% calcium).

A key issue of dietary calcium intake is not only its quantity but also quality in terms of bioavailability. Calcium bioavailability can be assessed using distinct approaches [26]. Here, we used a murine model to compare (a) nano-size fish bone particles from salmon frames with micro-size fish bone particles and (b) nano- and micro-size fish bone particles from salmon frames with calcium carbonate. The former evaluates the effect of particle size reduction on calcium bioavailability, and the latter is a comparison of fish bone particles from salmon frames with a commonly used calcium compound in supplementation, fortification, and the food industry. In addition, the effects were evaluated at a dietary level set at calcium requirement and at a higher one that was compatible with supplementation or fortification.

We defined calcium bioavailability as the percent of calcium in femur bone because it reflects both absorption and retention. The main results in this study were that salmon fish bone particle size reduction from 10 µm to 0.25 µm has a significant and positive effect on calcium bioavailability. This effect is observed at the requirement level, and it is much clearer at high dietary calcium levels. In addition, both micro- and nano-size salmon fish bone particles presented better calcium bioavailability figures than calcium carbonate. Figure 4 summarizes the main findings. The results expressed in this figure could be interpreted as calcium bioavailability from nano-size salmon fish bone being 15% and 20% higher than calcium carbonate at the calcium requirement and high level, respectively. Instead, the calcium bioavailability from micro-size salmon fish bone is only 3% and 8% higher than calcium carbonate at the calcium requirement and high level, respectively.

Calcium in fish bones, along with phosphorus, forms hydroxyapatite crystals. This compound has very low solubility, which may limit its absorption. Hydroxyapatite solubilization can be achieved by chemical, enzymatic, and mechanical methods; among the latter, particle size reduction stands out [8]. Thus, Li et al. [27] reported that after nano-milling, the crystallinity of hydroxyapatite from fish bone decreased and solubility increased. Two studies in murine models in Malaysia showed increased calcium absorption in nano-size calcium citrate and carbonate-fortified milk [28,29]. Our results, with similar particle size, provide evidence of increasing calcium bioavailability as a result of salmon fish bone particle size reduction from 10 µm to 0.25 µm. Furthermore, fish bone nanoparticles presented better bioavailability results than calcium carbonate, a product with a particle size of 0.75 µm.

There is a large body of evidence comparing calcium bioavailability using distinct compounds, such as carbonate, citrate, ascorbate, acetate, and fumarate, among others. Results are varied, although it seems consistent that citrate is equally or slightly better absorbed than the rest of the salts [30,31,32,33,34,35,36]. However, when cost is also considered, calcium carbonate presents some advantages [37]. Probably, that is the reason why the majority of supplementation or fortification interventions choose calcium carbonate. We used calcium carbonate as a control in our study for comparative purposes. Our findings are promising because both salmon fish bone microparticles and nanoparticles presented better calcium bioavailability results than calcium carbonate, allowing them to be considered as alternative calcium sources for further incorporation in a wide variety of foods.

As mentioned earlier, approximately 50% of fish industry products are by-products or waste [5]. In a current global context in which the fish industry must be aware of the need to assure long-term sustainable development that involves both people’s health and environmental protection while being economically competitive, contributions like those generated in this study may offer interesting new alternatives to reducing waste production, on the one hand, and encourage the development of new products for further use in the food industry, on the other. Indeed, additional information is needed for the full use of salmon fish bone nanoparticles, e.g., through sensory assessments of newly developed foods with these ingredients and economic evaluations. Although these aspects are beyond the scope of this study, it is possible to speculate that the positive results obtained in the experimental phase could lead to the development of new food products incorporating salmon fish nanoparticles, which provide alternatives to increase calcium intakes in populations that are not able or willing to consume milk products, a major dietary source of calcium.

Among the limitations of this study is that findings in a murine model must be interpreted as a first approximation to what may occur when humans are fed foods with salmon fish bone as a calcium source. Furthermore, it must be emphasized that this study only aimed to characterize salmon fish bone as an ingredient for further use in foods. Certainly, the final effect will depend on the food matrix to be incorporated and the host characteristics. This aspect is crucial, as reviewed by Shkembi et al. [38].

## 5. Conclusions

In this study, it was possible to produce fish bone microparticles and nanoparticles, with a substantial calcium content, from waste products of the salmon industry. Salmon fish bone nanoparticles present higher calcium bioavailability than salmon fish microparticles, and both, in turn, have better bioavailability than calcium carbonate. This warrants future studies of calcium supplementation or food products with these ingredients as fortification agents, as viable alternatives to common calcium sources.

## Figures and Tables

**Table 1:** Body weight, food intake, and femur weight and length during 21 days of dietary treatment with diets varying in calcium source and concentration.

Group	Initial Weight (g)	Final Weight (g)	Food Intake (g/d)	Femur Weight (mg)	Femur Length (mm)
CN 0.5 (*n* = 8)	16.6 ± 2.8 ^a^	25.5 ± 2.8 ^a^	7.0 ± 0.5 ^a^	48.9 ± 8.3 ^a^	14.3 ± 0.5 ^abc^
CN 1.0 (*n* = 8)	15.2 ± 2.1 ^a^	24.9 ± 3.0 ^a^	6.0 ± 0.8 ^b^	55.1 ± 8.1 ^ab^	14.1 ± 0.5 ^a^
MP 0.5 (*n* = 7)	20.2 ± 1.6 ^b^	26.0 ± 2.5 ^ab^	6.1 ± 0.3 ^b^	62.3 ± 11.8 ^bc^	14.8 ± 0.4 ^bc^
MP 1.0 (*n* = 9)	20.2 ± 2.1 ^b^	27.2 ± 2.5 ^abc^	6.2 ± 0.2 ^b^	61.6 ± 5.3 ^abc^	14.7 ± 0.2 ^abc^
NP 0.5 (*n* = 8)	24.7 ± 1.6 ^c^	30.2 ± 1.0 ^c^	6.2 ± 0.4 ^b^	75.7 ± 9.6 ^d^	15.4 ± 0.4 ^d^
NP 1.0 (*n* = 8)	23.9 ± 1.6 ^c^	29.6 ± 3.5 ^bc^	6.6 ± 0.1 ^ab^	62.7 ± 4.9 ^c^	14.9 ± 0.2 ^cd^

Values are mean ± SD. Statistical analysis by one-way ANOVA. Different superscripts in each column are statistically significant according to Tukey’s post hoc test (*p* < 0.05).

## Data Availability

The data presented in this study are available on request from the corresponding author. The data are not publicly available due to the unavailability of a public repository at the Department of Nutrition, Faculty of Medicine, University of Chile.
